# Fish Freshness Indicator for Sensing Fish Quality during Storage

**DOI:** 10.3390/foods12091801

**Published:** 2023-04-26

**Authors:** Do-Yeong Kim, Sung-Woo Park, Han-Seung Shin

**Affiliations:** Department of Food Science and Biotechnology, Dongguk University-Seoul, 32, Dongguk-ro, Ilsandong-gu, Goyang-si 10326, Gyeonggi-do, Republic of Korea; dykimm@dongguk.edu (D.-Y.K.); bigpso@naver.com (S.-W.P.)

**Keywords:** freshness gas indicator, intelligent packaging, fish spoilage, food quality

## Abstract

This study aims to develop a freshness indicator for fish products that changes color to indicate ammonia among volatile base compounds (TVB-N) generated during storage. Through an optimization experiment, we observed the indicator’s color change relative to the ammonia concentration standard, finally selecting cresol red and bromocresol purple for the indicator mixture. In addition, eco-DEHCH and Breathron film were applied to the freshness indicator, considering environmental and economic values. For the storage experiment, Chub mackerel (*Scomber japonicus*), Spanish mackerel (*Scomberomorus niphonius*), and Largehead hairtail (*Trichiurus lepturus*) samples were stored at three different temperatures (4, 10, and 20 °C) for seven days, and we measured pH, TVB-N, total bacterial count, and ammonia content every 24 h. The pH-sensitive sensors’ color changes monitor amine release, especially ammonia, from decomposing fish. The chromatic parameter ∆E value increased significantly with fish product storage periods. We confirmed that when the freshness limit and bacterial spoilage level were reached, the color of the indicator changed from yellow to black and sequentially changed to purple as the storage period increased. Therefore, a developed freshness indicator can be used for determining the quality of fish products quickly and non-destructively by reflecting the freshness and spoilage degree of fish products during storage.

## 1. Introduction

Packaging is crucial for the food industry’s quality and safety. Smart packaging is an easy and straightforward method for determining fish freshness after packaging [1]. Smart sensors attached to fish packaging represent innovative technology for monitoring product freshness and improving shelf life, food safety, and potential risks [2]. Incorporating a freshness indicator that can detect internal and external product changes with smart packaging further extends storage information capabilities by providing food quality and safety information [3]. In addition, they can help consumers intuitively and scientifically judge fish quality by supplying physiological change or microbial growth information [4].

Gas-sensitive array sensors offer several advantages, including high-speed recognition rates, no precise instrument requirements, and a broad scope for analyzing food [5]. The developed sensor is amine-sensitive and efficiently analyzes fish spoilage by measuring amine concentrations. The freshness of fish is reduced by physicochemical and microbiological changes. Microorganisms can be transformed into small compounds, such as free amino acids, through proteolytic activity [6]. In addition, amines such as ammonia, trimethylamine (TMA), and dimethylamine (DMA) produce odors during protein decomposition [7]. However, judging freshness by smell is difficult and unreliable, so an objective evaluation method is needed [8]. Traditional fish freshness evaluations include chemical analytical, microbiological, pH, and total volatile base nitrogen (TVB-N) methods; however, these methods are time-consuming and non-economic [9]. Compared to traditional evaluation methods, the pH-sensitive gas indicator is more efficient at evaluating fish freshness.

Freshness indicators respond to metabolites such as volatile base nitrogen from spoiled fish and monitor metabolite content through color-changing pH-sensitive sensors [10]. Polytetrafluoroethylene (PTFE) and polyvinylidene fluoride (PVDF) membranes were used as gas permeability and hydrophobic filters to block moisture from fish products and indicate freshness [11]. However, these membrane filters are unsuitable for the fish product industry because they are made of high-cost materials. Therefore, an economical and eco-friendly substrate is necessary. Breathron, a gas-permeable hydrophobic film, has a low cost, high gas permeability, and better moisture barrier porosity than other materials, blocking moisture from fish products and permeating volatile gases.

This study aims to develop a freshness indicator that non-destructively and economically determines fish product freshness and to evaluate its performance as a freshness indicator for the quality of fish involved, including Chub mackerel, Spanish mackerel, and Largehead hairtail, under three different temperature conditions (4, 10, and 20 °C) for seven days.

## 2. Materials and Methods

### 2.1. Materials

Cresol red, bromocresol purple, bromocresol green, and bromothymol blue were obtained from Sigma-Aldrich (St. Louis, MO, USA). Acetone and 2-butanone were obtained from Junsei Chemical Co. (Tokyo, Japan), and eco-DEHCH was obtained from Hanwha Solution Corp. (Seoul, Republic of Korea). Industrial filter paper (HC-50), polytetrafluoroethylene (PTFE), and polyvinylidene fluoride (PVDF) as gas-permeable membranes were acquired from Hyundai Micro., Ltd. (Seoul, Republic of Korea). Breathron was procured from Nitto Denko Corp. (Osaka, Japan). Cellulose acetate, ammonia solution 30% (*w*/*v*), potassium carbonate, anhydrous 99.5% (*w*/*v*), sodium hydroxide, 0.01 N H_2_SO_4_, and 0.01 N NaOH were purchased from Samchun Chemical Co. (Seoul, Republic of Korea). All other chemicals were analytical grade.

### 2.2. Sample Preparation

Chub mackerel (*Scomber japonicus*), Spanish mackerel (*Scomberomorus niphonius*), and Largehead hairtail (*Trichiurus lepturus*) fillets of different sizes, weights (150–200 g), and bones were purchased at a local market in Seoul, Republic of Korea, and transported in an ice box with a cooling pack to the laboratory. Each fresh individual fillet from the same containers was cut to 100 g and stored in a polypropylene container. Then, sample containers were sealed with polyethylene film using a packaging machine and kept at 4 °C for use immediately in the spoilage monitoring experiment.

### 2.3. Freshness Gas Indicator Development

**Figure 1:**
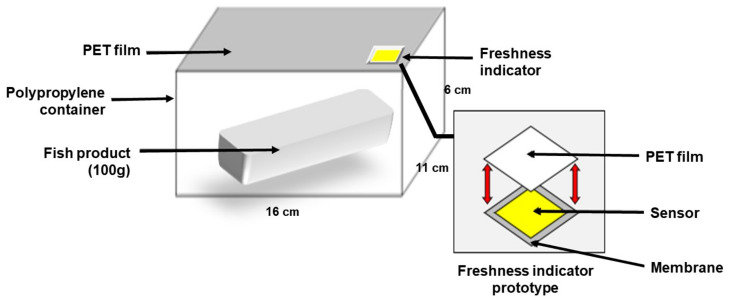
Schematic design of the freshness indicator for fish products.

40 mL of acetone and 2-butanone in a 1:1 ratio in a flask. A 1.2 g sample of cellulose acetate was dissolved at room temperature with a magnetic stirrer bar for an hour [12]. After complete dissolution, 0.3 mL of eco-DEHCH was added. Next, 8 mg of cresol red and 32 mg of bromocresol purple were added to the mixture and dissolved for an hour. The industrial filter paper (HC-50) was coated with the prepared mixture solution for 30 min, dried in a dark room for 2 h, and cut to 1.5 × 1.5 cm. This filter paper was attached to the gas-permeable and water-resistant film using double-sided tape (2 × 2 cm). The polyethylene terephthalate (PET) film is attached to it to protect the sensor from the external environment. Figure 1 organizes the prototype freshness gas indicator’s structural characteristics.

### 2.4. Chromaticity Analysis

#### 2.4.1. Polymer Matrix Solution Type and Concentration Differences

The gas-sensitive materials used included three different pH concentration indicators: bromocresol purple (P), bromocresol green (G), and bromothymol blue (B). When these three pH indicators were added to the polymer matrix solution, 8 mg, 20 mg, and 32 mg were added based on cresol red (C). For the spoilage simulation, nine freshness indicator-coated filter papers were prepared for each ratio (2:8, 5:5, 8:2). Experiments consisted of six different ammonia concentrations (0, 15, 30, 50, 100, and 200 mg/L (*w*/*v*)) by diluting a standard ammonia solution (Junsei Chemical Co., Tokyo, Japan) to simulate fish spoilage. The standard ammonia solution was placed in a polypropylene (PP) packaging container (16 cm × 11 cm × 6 cm), and double-sided tape attached the freshness indicator to the container’s headspace. The freshness indicator reacted at room temperature for 4 h for complete ammonia gas exposure. Then the freshness indicator’s chromatic changes were monitored by a colorimeter CR-20 (Konica Minolta, Tokyo, Japan). All measurements were conducted on three pH indicators in three different packages. The Hunter color value indicates that the chromatic parameter L (lightness) ranges from black to white, a (redness) ranges from red to green, and b displays a yellowness from yellow to blue. The color difference (∆E) was calculated using the following equation in relation to the color of the paper with 0 mg/L of ammonia:

$$\Delta E=\sqrt{{\left(L-L^{\prime }\right)}^{2}+{\left(a-a^{\prime }\right)}^{2}+{\left(b-b^{\prime }\right)}^{2}}$$

#### 2.4.2. Freshness Indicator’s Different Substrate Response Sensitivities

The components blocking moisture from fish products and permeating volatile base nitrogen were manufactured using hydrophobic PVDF-D, PTFE-D, and Breathron. The chromatic value evaluated the freshness indicators’ sensitivity.

### 2.5. pH Measurement

A 5 g sample of each fish (Chub mackerel, Spanish mackerel, and Largehead hairtail) was homogenized with 20 mL of distilled water, and the pH of suspensions was measured using a pH meter (Orion 3-star Meter; Thermo Fisher Scientific, Inc., Waltham, MA, USA) at three different storage temperatures (4, 10, and 20 °C) [13]. The pH meter was calibrated using the pH buffer solution (4.00 ± 0.01, 7.00 ± 0.01, 10.00 ± 0.01). All measurements were conducted in triplicate.

### 2.6. Total Volatile Basic Nitrogen (TVB-N) Content Measurement

TVB-N content was measured using the Conway micro-diffusion technique according to a previously reported method [14]. 5 g of fish fillets and 20 mL of distilled water were put in a 50 mL conical tube and homogenized at 200 rpm for 2 min using a homogenizer (HG-15D, Daihan Scientific, Seoul, Republic of Korea). The mixture was filtered through Whatman No.1 filter paper for 10 min. A 1 mL sample of filtrate and 1 mL sample of saturated K_2_CO_3_ solution were put in the outer space of the Conway dish. Then, 1 mL of 0.01 N H_2_SO_4_ solution was put in the inner space of the Conway dish. The sealed Conway dish was stored in a BOD incubator at 25 °C for an hour. After incubation, 1 mL of Brunswik regent (0.2 g methyl red and 0.1 g methylene blue were dissolved in 100 mL ethanol) was added to the inner space and titrated with 0.01 N NaOH. The following equation calculates fish fillets’ TVB-N content:

$$\mathrm{TVB}-N\;(\mathrm{mg}/100\;g)=0.14\times \frac{\left(b-a\right)\times f}{W}\times 100\times d$$

where b is the blank titration volume (distilled water was used instead of 0.01 N NaOH), a is the titration volume using 0.01 N NaOH, f is the 0.01 N NaOH standard factor, d is the dilution factor, and W is the fish fillet sample weight (g). Each sample was measured three times.

### 2.7. Microbial Analysis

Fish products were kept on ice at <4 °C refrigeration temperatures until division and analysis. Microbial analysis was conducted using the standard AOAC approval method in the FDA Bacterial Analysis Manual [15]. 25 g samples from each fish product were prepared at refrigeration temperature, transferred to filter stomacher bags, and mixed with 225 mL of sterile 0.1% peptone water (Difco, Detroit, MI, USA). The mixture was homogenized for 3 min using the stomacher (Bag Mixer 400, Interscience, St. Nom, France), and then the homogenized sample was serially diluted with sterile peptone water. Petrifilm^TM^ aerobic count plates (3M, St. Paul, MN, USA) were inoculated with diluted samples (1 mL each) and incubated at 37 °C for 48 h. Total bacterial counts (TBC) were recorded as colony-forming units per gram (log CFU/g).

### 2.8. Ammonia Content Measurement

The HS-SPME analysis followed Bene’s previously reported method for detecting amines in fish [16], with some modifications. A finely homogenized fish sample (2 g) was extracted with 20 mL of 0.5 N HCl for 5 min, and the resulting solution was centrifuged at 4000× *g* rpm for 10 min. The supernatant (2 mL) was poured into a 22 mL vial equipped with a magnetic stirrer bar, and 1 mL of 40% NaOH (*w*/*v*) was added and stirred for 15 min. For headspace extraction, the SPME needle guide was inserted, and then 65 mm of polydimethylsiloxane-divinylbenzene (PDMS-DVB) fiber (Supelco Co., Bellefonte, PA, USA) was immediately inserted into the injector port for analysis. PDMS-DVB fiber is the most suitable SPME fiber for measuring volatile nitrogen contents [17], with SPME as a simple, efficient, and solvent-free analysis method.

Gas chromatography-flame ionization detection (GC-FID) was performed with an Agilent 7890 instrument equipped with an HP-1 column (30 m × 0.32 mm × 0.25 μm; J&W Scientific, Folsom, CA, USA). Injector and detector temperatures were set at 200 and 250 °C, respectively. Splitless time was 1 min, and a desorption time of 4 min at 250 °C was enough for quantitative ammonia desorption. Nitrogen was used as the carrier gas at a 10 mL/min flow rate (constant flow). For FID, air and hydrogen were used at a 400 mL/min and 40 mL/min flow rate, respectively. The column temperature was held at 50 °C for 5 min, then increased by 10 °C/min to 120 °C and maintained for 20 min.

Quantitative analyses used standard ammonia products, and working solutions were prepared with 15, 50, 100, 200, 500, and 1000 mg/L concentrations diluted with GC-grade water. Six concentrations were calibrated, and R^2^ expressed a high 0.992 linearity accuracy. Detection and quantification limit calculations were 5.08 and 15.4, respectively. The 80.00% to 120.00% recovery was calculated using the peak area. Fish products were stored at three different temperatures (4, 10, and 20 °C) for seven days, and we measured ammonia content every 24 h.

### 2.9. Monitoring the Freshness Indicator’s Fish Spoilage and Chromaticity Change

We weighed 100 g of fish fillets in polypropylene containers. Then, the developed freshness indicator was attached to the packaging container’s headspace, and the spoilage trial was conducted in the biochemical oxygen demand (BOD) incubator. A smartphone camera (Galaxy 10e; Samsung Electronics Co., Ltd., Seoul, Republic of Korea) documented the freshness indicator’s color change, and photographs of the color change were taken at 24-h intervals. Next, the colorimeter (CR-20; Konica Minolta, Tokyo, Japan) monitored the freshness indicator’s chromatic change.

### 2.10. Statistical Analysis

All experiments were performed in triplicate (n = 3) and analyzed using IBM SPSS Statistics 20.0 (IBM, Armonk, NY, USA). Values were presented as mean ± standard deviation (SD), and a one-way analysis of variance (ANOVA) was conducted. The Student-Newman-Keuls test was used for appropriate comparison, with significance defined as *p* < 0.05 for ANOVA.

## 3. Results and Discussion

### 3.1. Freshness Indicator Optimization from Chromaticity

**Figure 2:**
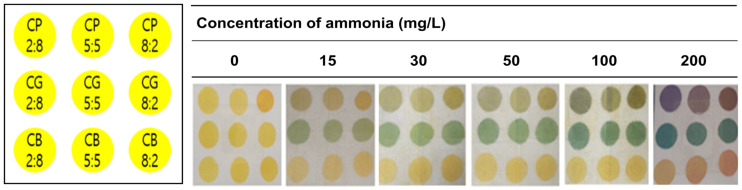
The color changes of the array sensor according to the ammonia concentration.

Declining fish freshness generates gas components, and humans are slow in detecting these gas composition changes, but freshness indicators can trace this process [18]. Therefore, the indicator’s color change must be identifiable even during initial fish spoilage, and there should also be a sequential change in fish states. In fish meat, the pH value increases from 6.2 to 7.5 during prolonged storage [19]. Figure 2 indicates the color change when a freshness indicator coated with nine different dye solution ratios is exposed to six standard ammonia concentrations (0, 15, 30, 50, 100, and 200 mg/L). We monitored nine freshness color change indicators following ammonia gas exposure. Cresol red-bromocresol purple (CP) pH indicators were noted as yellow at 15 mg/L ammonia and consistently changed from yellow to purple as ammonia concentration increased. Cresol red-bromocresol green (CG) pH indicators displayed bright green during initial ammonia exposure and were continuously monitored as ammonia concentration increased.

However, cresol red-bromothymol blue (CB)-coated pH indicators did not change color regardless of ammonia exposure interval. The CG freshness indicator changes from bright yellow to green in two stages depending on the ammonia content, while the CP freshness indicator changes color in three stages: yellow, black, and purple. However, naked-eye recognition and quantification are complex [20]. Visual inspection failed to distinguish any difference between 15 and 30, or between 30 and 50 mg/L samples of the CP freshness indicator. Therefore, the chromaticity value of the freshness indicator containing CP measured varying ammonia content, as summarized in Table 1. There were significant differences (*p* < 0.05) in the chromatic values of the samples, with the values of L, a, and b changing from 76.4 to 60.8, from 14.3 to −1.5, and from 28.4 to 12.2, respectively. In addition, ∆E (chromaticity change) value sequentially increased from 0 to 60.2, as the originally yellow freshness indicators turned purple (Figure 2). These results confirm that the CP pH indicator can efficiently monitor fish product ammonia levels.

The freshness indicator’s sensor is sensitive to pH changes, so moisture generated during fish product storage must be blocked. Hydrophobic membranes and films are utilized to block moisture from sensors. The porous hydrophobic membranes PTFE-D and PVDF-D have high gas permeability and excellent moisture barrier characteristics. Since the indicator’s sensor is sensitive to moisture and changes in the external environment during marine product storage, a hydrophobic and gas-permeable membrane was selected [11]. Due to their high cost, gas-sensitive reverse-phase silica plates and substrate materials, such as PVDF, are challenging to incorporate into the food industry [18]. The Breathron film is economical compared to these two membranes. The film has a triple PE porous film, adhesive layer, and nonwoven fabric structure that blocks moisture generated from fish products and has gas permeability (Figure 1).

We simulated spoilage conditions to evaluate the sensitivity of the freshness indicator’s three materials to standard ammonia products. Table 2 conveys the chromaticity exposure results of six standard ammonia concentrations. The PTFE-D membrane freshness indicator exhibited less chromatic change when the ammonia concentration increased from 100 to 200 mg/L, and the PVDF-D membrane displayed the best sensitivity for detecting ammonia among the three membranes. This result showed that PVDF-D membrane can be suitable for fish products, except when considering economic aspects.

On the other hand, the Breathron film freshness indicator constantly changed as chromaticity fluctuation simultaneously increased alongside ammonia concentration elevation. A number of techniques have been proposed to investigate the fish’s freshness, such as the detection of chemical changes and instrumental methods like the electronic nose (E-nose). E-nose systems are especially attractive in the food industry, but they are challenging to eliminate interference resulting from changes in humidity [21]. However, the developed freshness indicator using Breathron film in this study has the advantage that it is not greatly affected by humidity changes due to its hydrophobicity. When considering hydrophobicity and gas permeability, the Breathron film was remarkably inexpensive, and sequential color changes were readily observed in ammonia exposure and fish storage experiments.

### 3.2. Chemical and Microbiological Analysis of Stored Fish Samples

#### 3.2.1. pH Measurement

**Figure 3:**
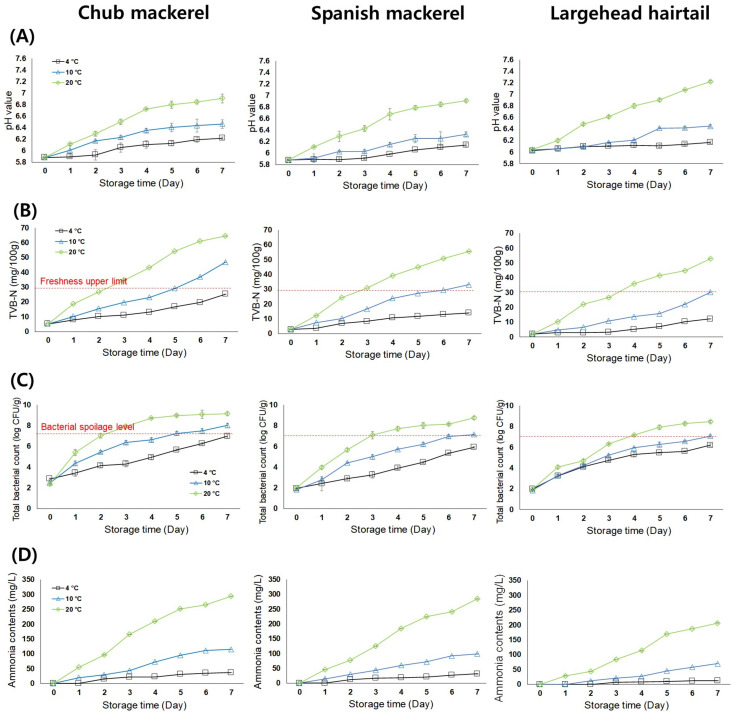
pH (**A**), total volatile base nitrogen (TVB-N) (**B**), total bacterial counts (**C**), and ammonia content (**D**) of fish samples stored at 4 °C, 10 °C, and 20 °C for 7 days.

Figure 3A illustrates pH changes for fish fillets at 4 °C, 10 °C, and 20 °C for seven days, where pH values gradually increased during storage. Fresh Chub and Spanish mackerels exhibited a 5.7 to 5.8 pH range, whereas fresh Largehead hairtail’s pH was 6.06. In other studies, the fresh hairtail’s initial pH was 6.4, reaching 6.7 on day 7 [22]. The pH slightly decreased at storage onset but increased over time. Lactic acid accumulation causes the initial pH to lower, gradually increasing proteolysis production’s resulting alkalization [7]. Fresh Largehead hairtail’s initial pH was relatively high, thus maintaining a relatively high pH regardless of freshness. The Chub and Spanish mackerel pH rapidly increased at 20 °C storage temperature on day 7, reaching 6.91 and 6.90, respectively.

The Largehead hairtail’s pH remained relatively higher than that of the Chub and Spanish mackerel at all storage temperatures. Previous studies elucidate that pH is affected by TVB-N accumulation in a fish’s microbial activity [23]. TMA and DMA formation generally increase fish pH during storage [24]; thus, pH escalation is associated with storage spoilage. However, initial pH levels decreased slightly for fish products stored at refrigerated temperatures. The initial pH decrease occurs due to lactate accumulation, and the fish’s post-protein proteolytic product release increases pH to alkalization [25].

#### 3.2.2. Total Volatile Basic Nitrogen (TVB-N) Analysis

**Figure 4:**
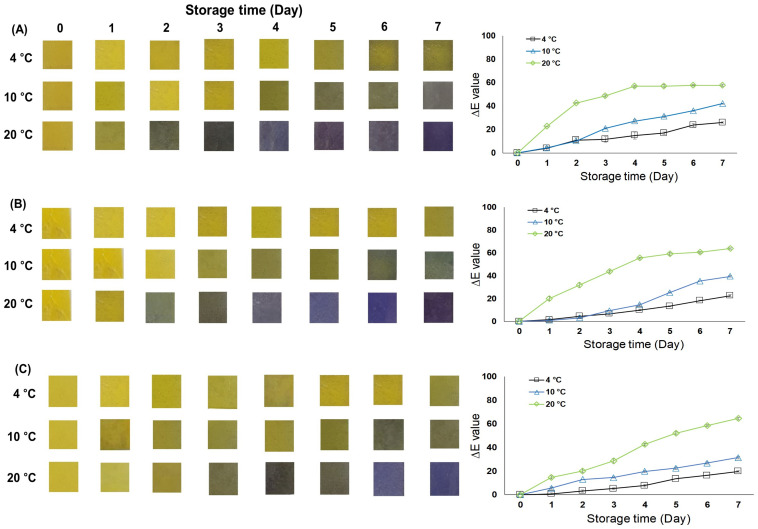
Changes in color of the freshness indicator (**left**) and ∆E value (**right**) during storage experiments at 4 °C, 10 °C, and 20 °C for 7 days: (**A**) Chub mackerel; (**B**) Spanish mackerel; (**C**) Largehead hairtail.

Figure 3B and Figure 4 portray the TVB-N content and sensor color changes when stored at 20 °C for seven days. Sensor chromaticity changed from black to purple when TVB-N content exceeded the upper freshness limit. TVB-N analysis is a chemical evaluation of fish product freshness. After the fish dies, microorganisms break down proteins to produce nitrogen compounds such as amines. Fresh fish TVB-N content ranges from 5–10 mg/100 g until reaching the upper 30 mg/100 g limit [22]. Fresh fish fillet TVB-N content is 1 to 5 mg/100 g. The TVB-N content of the three fish products at a 4 °C refrigeration temperature did not reach the upper freshness limit during seven days of storage.

In a previous study, bass reached 13 mg/100 g on day 6 and 20 mg/100 g on day 9 when stored at 4 °C [18]. Bass is a white fish, and its share increased similarly to Largehead hairtail’s increased TVB-N. Chub and Spanish mackerel reached the upper freshness limit of 10 °C on day 6; Largehead hairtail reached this limit on day 7. At the standard 20 °C temperature, Chub and Spanish mackerel TVB-N content increased rapidly, reaching the upper freshness limit on day 3, which the Largehead hairtail reached on day 4. When fish products’ TVB-N content reached the upper freshness limit, the freshness indicator sensor’s color changed to black.

#### 3.2.3. Microbial Analysis

Certain spoilage microorganisms exist in small amounts upon storage onset but increase over time, proliferating and producing metabolites such as TVB-N and CO_2_ that change color, smell, taste, and texture and cause sensory rejection [24,26]. Microbial growth was evaluated by monitoring TBC for seven days at three temperatures (4, 10, and 20 °C), and the bacterial spoilage threshold was log 7.0 CFU/g [27]. As shown in Figure 3C, the initial fresh fish product TBC was 2.02 to 2.8 log CFU/g. At 4 °C refrigerated temperatures, the three fish products did not reach the bacterial spoilage threshold even on day 7. Another study conducted a 4 °C salmon storage experiment for nine days, and the TBC reached the bacterial spoilage threshold on day 6 [28]. Chub and Spanish mackerel reached the bacterial spoilage threshold on days 7 and 8. At a 10 °C storage temperature, the Chub and Spanish mackerel reached this threshold on days 5 (7.07 log CFU/g) and 6 (7.12 log CFU/g). On the other hand, the Largehead hairtail did not reach the bacterial spoilage threshold until day 7 (7.01 log CFU/g). At the standard 20 °C, the three fish products’ TBC increased rapidly, with the Chub and Spanish mackerel reaching the bacterial spoilage threshold by day 3 (7.82 log CFU/g, 7.09 log CFU/g). However, the Largehead hairtail did not reach this until day 4 (7.25 log CFU/g).

Concurrently with the indicator’s chromaticity change in Figure 4, the indicator’s color also changed to black, consistent with when the bacterial spoilage threshold was reached. Figure 3C and Figure 4 convey the APC growth and sensor color changes when stored at 20 °C for seven days. The sensor chromaticity changed from black to purple when APC growth exceeded the bacterial spoilage threshold. Therefore, the developed freshness indicator can efficiently denote food hygiene management and fish freshness determination.

#### 3.2.4. Ammonia Content Analysis

Figure 3D shows the ammonia content of fish samples stored at three different temperatures (4, 10, and 20 °C) for seven days. No ammonia was detected in fresh fish products. It is generally known that the ideal storage temperature for fish products to maintain freshness is 4 °C or lower. Therefore, there are no significant changes in ammonia concentration at low temperatures, and it may seem difficult to see the color changes of the indicator during storage (Figure 4). However, a significant change in ammonia concentration was observed in all samples as the storage temperature increased. Notably, ammonia production rapidly surged at 20 °C, especially in Chub and Spanish mackerel. On the other hand, in the trial with Largehead hairtail, ammonia production slowly increased at all storage temperatures. It is suggested that developed indicators can detect the degree of deterioration that can occur during refrigerated storage and distribution processes.

### 3.3. Indicator Response during Storage

Our developed freshness indicator was attached to the fish product’s packaging headspace to monitor the indicator’s color change. The volatile base nitrogen compound continuously released from the fish product accumulated in the packaging headspace. Figure 4 presents the freshness indicator’s image during fish fillet storage. At 4 °C refrigeration, the indicator chromaticity changed very slowly. The freshness indicator’s color rapidly changed at 20 °C, turning black on day 3 in the Chub mackerel. In the trial with Largehead hairtail, the color of the indicator changed to black a day later, on day 4. This result relates to microbiological and chemical upper limits and can predict product freshness at each step.

Figure 4 summarizes the freshness indicator’s colorimetric analysis. At 20 °C storage, the three fish products’ ∆E value rapidly escalated. In particular, mackerel ∆E values of 22.59, 42.58, 48.70, and 56.86 indicated a substantial change during the early storage stages. After four days, the indicator’s ∆E value scarcely changed. The E value increased slightly when the freshness indicator color changed to purple. In other studies, the freshness indicator using bromocresol purple also changed to purple as the storage period passed [29]. The human eye can readily detect a color change with an ∆E value of 5 or more and noticeably detect color changes at 12 or more [30]. When stored in a packaging container, our developed freshness indicator can non-destructively identify fish freshness.

## 4. Conclusions

The freshness indicator developed in this study can non-destructively identify the freshness of fish stored in a packaging container. The pH changes relative to ammonia concentrations in the packaging container’s headspace, changing the freshness indicator’s color. Chemical and microbiological analyses assessed fish freshness and evaluated the developed freshness indicator’s color change. The freshness indicator’s color changed from yellow to black when the freshness limit and bacterial spoilage range were exceeded and sequentially changed to purple as the storage period increased. Freshness indicators incorporated two pH indicators: bromocresol purple, cresol red, eco-DEHC, and Breathron film. These indicators’ pH ranges highly relate to fish freshness, and the non-phthalate eco-DEHCH used as a plasticizer in the manufactured dye mixture is eco-friendly. Considering hydrophobicity and gas permeability, the Breathron film was remarkably inexpensive, and sequential ammonia-based color changes were observed during exposure and storage experiments. A developed freshness indicator can effectively determine freshness during fish distribution and storage. In addition, it can help to ensure food safety by providing consumers with an easy way to determine if a fish product is fresh or spoiled, which could prevent foodborne illness. However, further studies are needed to understand the correlation between the color of the sensors and target indicators such as pH, TVB-N, and TBC during storage and to validate them with a larger sample size and various fish species to ensure that they can accurately predict freshness and spoilage levels in all types of fish.

## Figures and Tables

**Table 1:** Chromatic values of the cresol red-bromocresol purple (CP, 2:8) pH indicator in response to ammonia standard solution.

	Concentration of Ammonia (mg/L)					
	0	15	30	50	100	200
∆E	0 ^a^	22.3 ± 1.9 ^b^	40.3 ± 3.4 ^c^	46.3 ± 1.5 ^c^	54.9 ± 1.7 ^d^	60.2 ± 1.2 ^e^
L	80.4 ± 1.9 ^f^	76.4 ± 1.1 ^e^	63.4 ± 2.7 ^d^	60.8 ± 1.6 ^c^	54.7 ± 1.7 ^b^	51.1 ± 1.0 ^a^
a	16.0 ± 3.1 ^d^	14.3 ± 1.4 ^c^	−3.4 ± 4.1 ^a^	−1.5 ± 0.9 ^b^	1.6 ± 0.6 ^b^	0.1 ± 0.9 ^b^
b	50.3 ± 2.4 ^f^	28.4 ± 2.2 ^e^	19.4 ± 3.0 ^d^	12.2 ± 2.1 ^c^	4.0 ± 2.2 ^b^	0.2 ± 1.2 ^a^

Different letters in the same line indicate significant differences (*p* < 0.05). All values are the mean ± standard deviation of triplicate determinations.

**Table 2:** Chromatic values of freshness indicators responding to ammonia concentration.

Ammonia Concentration (mg/L)	∆E Value		
	PTFE-D	PVDF-D	Breathron
0	0 ^aA^	0 ^aA^	0 ^aA^
15	10.1 ± 1.4 ^bA^	19.6 ± 1.7 ^bC^	15.0 ± 1.1 ^bB^
30	27.1 ± 2.1 ^bA^	26.1 ± 3.3 ^bA^	23.9 ± 2.4 ^bA^
50	42.3 ± 4.6 ^cB^	32.3 ± 2.1 ^cA^	29.2 ± 1.7 ^cA^
100	51.3 ± 1.0 ^dB^	43.4 ± 3.4 ^dA^	38.7 ± 2.5 ^dA^
200	54.9 ± 0.6 ^eB^	48.1 ± 2.0 ^eA^	44.9 ± 1.3 ^eA^

Different letters (a–e) in the same column indicate significant differences (*p* < 0.05). Different letters (A–C) in the same line indicate significant differences (*p* < 0.05). All values are the mean ± standard deviation of triplicate determinations.

## Data Availability

The data presented in this study are available in the article.

## References

[1] Pacquit A., Lau K.T., McLaughlin H., Frisby J., Quilty B., Diamond D. (2006). Development of a volatile amine sensor for the monitoring of fish spoilage. Talanta.

[2] Mustafa F., Andreescu S. (2018). Chemical and Biological Sensors for Food-Quality Monitoring and Smart Packaging. Foods.

[3] Sohail M., Sun D.-W., Zhu Z. (2018). Recent developments in intelligent packaging for enhancing food quality and safety. Crit. Rev. Food Sci. Nutr..

[4] Ghaani M., Cozzolino C.A., Castelli G., Farris S. (2016). An overview of the intelligent packaging technologies in the food sector. Trends Food Sci. Technol..

[5] Xiao-wei H., Xiao-bo Z., Ji-yong S., Zhi-hua L., Jie-wen Z. (2018). Colorimetric sensor arrays based on chemo-responsive dyes for food odor visualization. Trends Food Sci. Technol..

[6] Grzyb A., Wolna-Maruwka A., Niewiadomska A. (2021). The Significance of Microbial Transformation of Nitrogen Compounds in the Light of Integrated Crop Management. Agronomy.

[7] Lee E.-J., Shin H.-S. (2019). Development of a freshness indicator for monitoring the quality of beef during storage. Food Sci. Biotechnol..

[8] Tonezzer M. (2021). Detection of Mackerel Fish Spoilage with a Gas Sensor Based on One Single SnO_2_ Nanowire. Chemosensors.

[9] Anderson J.S., Lall S.P., Anderson D.M., Mcniven M.A. (1993). Evaluation of Protein-Quality in Fish Meals by Chemical and Biological Assays. Aquaculture.

[10] Kuswandi B., Wicaksono Y., Abdullah A., Heng L.Y., Ahmad M. (2011). Smart packaging: Sensors for monitoring of food quality and safety. Sens. Instrum. Food Qual. Saf..

[11] Chun H.N., Kim B., Shin H.S. (2014). Evaluation of a freshness indicator for quality of fish products during storage. Food Sci. Biotechnol..

[12] Byrne L., Lau K.T., Diamond D. (2002). Monitoring of headspace total volatile basic nitrogen from selected fish species using reflectance spectroscopic measurements of pH sensitive films. Analyst.

[13] Lee G.-Y., Lee S., Shin H.-S. (2016). Evaluation of gas freshness indicator for determination of skate (*Raja kenojei*) quality during storage. Food Sci. Biotechnol..

[14] Kim M.-J., Shin H.-S. (2011). Effect of treatment with ozonated water on shelf life of refrigerated meat. Food Sci. Anim. Resour..

[15] Sarnoski P.J., O’Keefe S.F., Jahncke M.L., Mallikarjunan P., Flick G.J. (2010). Analysis of crab meat volatiles as possible spoilage indicators for blue crab (*Callinectes sapidus*) meat by gas chromatography—Mass spectrometry. Food Chem..

[16] Béné A., Hayman A., Reynard E., Luisier J., Villettaz J. (2001). A new method for the rapid determination of volatile substances: The SPME-direct method: Part II. Determination of the freshness of fish. Sens. Actuators B Chem..

[17] Loughran M., Diamond D. (2000). Monitoring of volatile bases in fish sample headspace using an acidochromic dye. Food Chem..

[18] Liu H.Y., Zhang Y.R., Huang L., Wang M.Y. (2022). A colorimetric gas-sensitive array sensor using filter paper for the analysis of fish freshness. Food Chem..

[19] Chan S.T., Yao M.W.Y., Wong Y.C., Wong T., Mok C.S., Sin D.W.M. (2006). Evaluation of chemical indicators for monitoring freshness of food and determination of volatile amines in fish by headspace solid-phase microextraction and gas chromatography-mass spectrometry. Eur. Food Res. Technol..

[20] Tilahun S., An H.S., Hwang I.G., Choi J.H., Baek M.W., Choi H.R., Park D.S., Jeong C.S. (2020). Prediction of α-solanine and α-chaconine in potato tubers from hunter color values and VIS/NIR spectra. J. Food Qual..

[21] Huang X., Xin J., Zhao J. (2011). A novel technique for rapid evaluation of fish freshness using colorimetric sensor array. J. Food Eng..

[22] Yu J.-W., Kim H.-J., Seol D.-E., Ko J.-Y., Kim S.-H., Yang J.-Y., Lee Y. (2019). Evaluation of Largehead Hairtail Trichiurus lepturus Freshness Using Sensory and Chemical Analyses. Korean J. Fish. Aquat. Sci..

[23] Castro P., Padrón J.C.P., Cansino M.J.C., Velázquez E.S., De Larriva R.M. (2006). Total volatile base nitrogen and its use to assess freshness in European sea bass stored in ice. Food Control.

[24] Gram L., Huss H.H. (1996). Microbiological spoilage of fish and fish products. Int. J. Food Microbiol..

[25] Florek M., Litwinczuk A., Skalecki P., Ryszkowska-Siwko M. (2007). Changes of physicochemical properties of bullocks and heifers meat during 14 days of ageing under vacuum. Pol. J. Food Nutr. Sci..

[26] Rukchon C., Nopwinyuwong A., Trevanich S., Jinkarn T., Suppakul P. (2014). Development of a food spoilage indicator for monitoring freshness of skinless chicken breast. Talanta.

[27] Pacquit A., Frisby J., Diamond D., Lau K.T., Farrell A., Quilty B., Diamond D. (2007). Development of a smart packaging for the monitoring of fish spoilage. Food Chem..

[28] Morsy M.K., Zór K., Kostesha N., Alstrøm T.S., Heiskanen A., El-Tanahi H., Sharoba A., Papkovsky D., Larsen J., Khalaf H. (2016). Development and validation of a colorimetric sensor array for fish spoilage monitoring. Food Control.

[29] Kim D., Lee S., Lee K., Baek S., Seo J. (2017). Development of a pH indicator composed of high moisture-absorbing materials for real-time monitoring of chicken breast freshness. Food Sci. Biotechnol..

[30] Ran R., Wang L., Su Y., He S., He B., Li C., Wang C., Liu Y., Chen S. (2021). Preparation of pH-indicator films based on soy protein isolate/bromothymol blue and methyl red for monitoring fresh-cut apple freshness. J. Food Sci..

